# *Solanum watneyi*, a new bush tomato species from the Northern Territory, Australia named for Mark Watney of the book and film “The Martian”

**DOI:** 10.3897/phytokeys.61.6995

**Published:** 2016-02-25

**Authors:** Christopher T. Martine, Emma S. Frawley, Jason T. Cantley, Ingrid E. Jordon-Thaden

**Affiliations:** 1 Department of Biology, Bucknell University, 1 Dent Drive, Lewisburg, PA, USA; 2 University and Jepson Herbaria, University of California, Berkeley, CA, USA

**Keywords:** Judbarra, Gregory National Park, Solanum eburneum, Solanum sp. Bullita, Mark Watney, Matt Damon, The Martian, andromonoecy, Andy Weir

## Abstract

A new species of andromonoecious *Solanum* from the Australian “bush tomato clade” of Solanum subgenus Leptostemonum is described. *Solanum watneyi* Martine & Frawley, **sp. nov.** is closely allied with *Solanum eburneum*, and is sympatric with it in parts of its range in the Northern Territory. The new species has been recognized as a variant of *Solanum eburneum* for decades, at times being referred to by local botanists as *Solanum* sp. “Bullita” because of its relative abundance in the vicinity of the Bullita Station area of Judbarra/Gregory National Park. Morphometric analyses show that *Solanum watneyi* differs statistically from *Solanum eburneum* in several key reproductive and vegetative characters and field observations suggest that the two sister species may represent a case of edaphic speciation. We provide morphometric evidence for the novelty of *Solanum watneyi*, a complete description, and cite specimens for both species.

## Introduction

Since David Symon’s monograph of Australian *Solanum* L. in 1981, numerous additional species and morphospecies have been described for the continent. This has been notably true for a set of “spiny solanums” from northern Australia defined by Symon (1981) as belonging to Solanum subgenus Leptostemonum Bitter section Melongena Bitter (e.g. Bean and Albrecht 2008, Barrett 2013, Brennan et al. 2006, Martine et al. 2011, 2013), a putatively natural group including the cultivated eggplant (*Solanum melongena* L.) and a number of other non-Australian species. Nine of the morphologically androdioecious – but functionally dioecious - spiny solanums known in Australia at that time were included by Symon in this group, as were a set of species that exhibit andromonoecy, with each inflorescence consisting of a basal hermaphroditic flower accompanied by numerous staminate flowers that rise above it in a distal cyme.

Later work employing molecular phylogenetics (Martine et al. 2006, 2009, Vorontsova et al. 2013) showed that section *Melongena* sensu Symon (1981) was polyphyletic. A set of Australian andromonoecious species, however (including *Solanum chippendalei* Symon, *Solanum diversiflorum* F. Muell. and *Solanum beaugleholei* Symon), represent a moderately supported grouping identified by Martine et al. (2006) as the “bush tomato clade” – a finding that supports inferences by Symon (1981), Whalen (1984) and Bean (2004) regarding their similarities and relatedness. One species inferred to be in this clade, *Solanum eburneum* Symon, was among the many new species described by Symon (1971) during his long career.

Apparently endemic to a small region of the Northern Territory around the East Baines River, *Solanum eburneum* appeared to Symon to be restricted to gray clay soils. Meanwhile, botanists collecting in surrounding areas, including Pete Latz (NT Herbarium, Alice Springs), had observed populations across a broader range that were recognizably distinct from the similar *Solanum eburneum*. A specimen sent by Latz to Symon in 1976 led the latter botanist to conclude that it matched “no very satisfactory name” used at the time and that he could “do no better than affin. *eburneum*” (excerpt from letter on specimen NTD9293, *P.K. Latz 5325*). Field identification was also sometimes challenging, especially along the Bullita Stock Route where the range of the apparently more widespread morphotype comes into contact with *Solanum eburneum* in Judbarra/Gregory National Park. Still, the morphotype was recognizable enough to be identified for years on collection labels as *Solanum* sp. “Bullita” (*Latz 12401*) in reference to Latz’s assignment of that identifier to the putative new taxon beginning in the early 1970s. Until now, however, no morphometric comparison between the two morphotypes has been undertaken.

In 2014, CTM and family surveyed the vicinity of the East Baines/Victoria Highway/Bullita Stock Route region for occurrences of *Solanum eburneum* and *Solanum* sp. “Bullita.” Seeds of typical *Solanum eburneum* (from the type locality as per Symon 1981) and *Solanum* sp. “Bullita” populations were collected and then grown at Bucknell University, where morphometric analyses were conducted on the two taxa in order to define the differences between them and to determine the novelty of *Solanum* sp. “Bullita.” We here describe this species as new, and contrast both its morphology and distribution with its close relative *Solanum eburneum*.

## Morphological comparisons

In early May 2014, populations of *Solanum eburneum* were visited along the Victoria Highway corridor between Timber Creek and the Northern Territory border with Western Australia, including the type locality (Symon 1981) at the intersection of the highway and the East Baines River. Herbarium specimens and mature fruits were collected, with seeds extracted, dried, and stored for future use. Similar populations identified as *Solanum* sp. “Bullita” were then collected in the same manner along the Bullita Stock Route in the Bullita section of Judbarra/Gregory National Park. Leaf material for each taxon was collected and dried in silica for subsequent DNA work (not included here).

**Figure 1.**
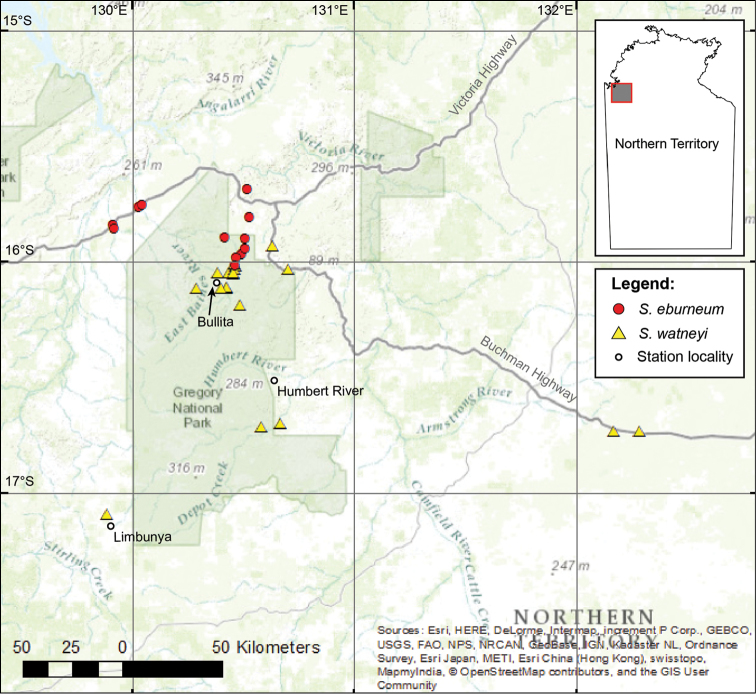
Distribution map of *Solanum watneyi* and *Solanum eburneum* based on accessions held by DNA, BUPL and CONN. Specimens of *Solanum eburneum* mapped are cited in Appendix 1.

To establish populations for morphometric analyses seeds were soaked for 24 hours in 1000-ppm gibberellic acid and sown in a controlled growth chamber environment at Bucknell University. They germinated at a rate of 70-90% in 2-3 weeks, with seedlings transferred to individual pots in our research greenhouse after establishment. All plants were grown in a common soil mix under identical horticultural conditions. Thirty-eight vegetative and reproductive characters were measured across developmental stages in 41 cultivated plants. These data were analyzed using ANOVA in the statistical software JMP-Pro 12 (SAS Institute Inc., Cary, North Carolina, USA) to define characters differing between the taxa. Character differences were corroborated and “field-truthed” by examination of herbarium specimens, including accessions held at the Northern Territory Herbarium, Palmerston (DNA) (See “specimens examined” and Appendix 1) from across the geographic range of each taxon (Figure 1). Characterization of trichomes is based on descriptions by Bean (2012) for *Solanum* sp. “Bullita” derived from the author’s examination of herbarium specimens.

## Results

The ANOVA analyses found that of 38 discrete vegetative and reproductive characters that were measured, 17 differed with statistical significance between *Solanum eburneum* and *Solanum watneyi* (Table 1). An additional set of qualitative characters also proved useful for drawing distinctions between the taxa (Table 2).

**Table 1.** Statistical comparison of characters, *Solanum watneyi* and *Solanum eburneum*. Single asterisk (*) indicates statistical significance with a 95-99% confidence interval; double asterisk (**) indicates statistical significance with >99% confidence interval. SD = standard deviation. All measurements in cm, except trichome density (per 0.5 cm leaf disk) and number of seeds per fruit.

	*Solanum eburneum*			*Solanum watneyi*			
Character	Average	SD	n	Average	SD	n	p-value
internode length	2.16	0.67	16	4.01	0.98	25	<.0001**
petiole length	2.68	0.80	16	3.36	3.36	25	<.0095**
stem prickle length	0.40	0.00	16	0.26	0.26	25	<.0001**
apical (upper 2-3 stems) leaf length	11.32	0.10	16	12.39	2.5	25	<.1372
apical leaf width	1.41	0.41	16	2.47	0.65	25	<.0001**
basal (lower 2-3 stems) leaf length	13.66	1.83	16	16.80	3.85	25	<.0061**
basal leaf width	2.03	0.88	16	3.97	1.15	25	<.0001**
male corolla diameter	3.46	0.31	16	4.01	0.62	25	<.0061**
hermaphrodite corolla diameter	4.03	0.35	16	4.75	0.58	25	<.0017**
fruit pedicel length	3.45	0.64	14	4.22	0.65	19	<.0341*
plant height	43.62	10.86	16	45.85	6.90	25	<.4165
leaf adaxial trichome density	174	59.00	24	105.00	21.46	25	<.0001**
leaf abaxial trichome density	194	26.00	24	133.00	27.81	28	<.0004**
fruit length	1.80	0.29	12	2.13	0.36	20	<.0058**
fruit width	2.20	0.38	12	1.89	0.31	20	<.0364*
fruit wall width	0.31	0.09	5	0.55	0.09	5	<.0038**
seeds per fruit	78	38.00	12	44.00	23.62	20	<.0031**

**Table 2.** Selected qualitative characters found to differ conspicuously between *Solanum eburneum* and *Solanum watneyi* sp. nov.

Character	*Solanum eburneum*	*Solanum watneyi*
habit	erect, compact	sprawling/lax, open
lobing of leaves	deep, numerous	± shallow (if present), few
corolla color	darker purple, ‘mauve’	lighter purple, ‘dusty purple’
corolla margins	more or less flat	wavy, undulating
fruit shape	± globose	± ellipsoidal
fruit color at maturity	white, ‘creamy’ without striping	yellow, ‘light lemon’ with light brown striping
fruit interior at maturity	liquid-filled	more or less dry
fruit firmness at maturity	soft, squishy	firm
fruit location	pendant from stems, but not on ground	pendant, on or near ground
seed color at maturity	black	light to dark brown

## Taxonomic treatment

### Solanum watneyi

Taxon classificationPlantaeSolanalesSolanaceae

Martine & Frawley sp. nov.

urn:lsid:ipni.org:names:77153383-1

Figs 2 , 3

#### Diagnosis.

*Solanum watneyi* is distinguished from *Solanum eburneum* by its weakly erect and sprawling habit, long internodes and fruiting pedicels, often scabrous dark green leaves, dusty purple corollas, and lemon-yellow thickly-walled ellipsoidal fruits often held on or near the ground.

#### Type.

AUSTRALIA. Northern Territory: Judbarra/Gregory National Park, Bullita Stock Route, 6 km north of Bullita Campground turnoff, 16°03.100"S, 130°27.201"E, 6 May 2014 (staminate and hermaphrodite flowers; fruit), *Christopher T. Martine and Rachel F. Martine 4065* (holotype: DNA; isotypes: BUPL, CONN)

#### Description.

Weakly erect to sprawling sub-shrub to 50–60 cm. Rhizomatous and apparently clonal. Stems slender, woody, often bending to ground, especially when weighted by fruits; initially single stemmed, with strong lateral branching beginning at ca. 5–10 cm; internode lengths on mature stems averaging about 4 cm. Overall plant aspect dark green to gray-green, with older stems eventually pea-green to yellow-brown; pubescent throughout with porrect stellate trichomes (stems, leaves, pedicels, calyces) with stalks 0–0.1 mm long, lateral rays 6–10, central ray (midpoint) 1–1.5 times as long as the lateral rays; pubescence of stems short and loose. Prickles sparse, straw-colored, straight, slightly widened at base, fine, 2–5 mm long, scattered on stems. Sympodial units difoliate, the leaves solitary or geminate. Mature leaves 12–16 cm × 2–6 cm, alternate, lanceolate-elliptic, with 6–8 pairs of primary veins; young leaves lighter green and gray-hairy but becoming dark green above, slightly paler beneath, both sides closely and densely stellate tomentose, older leaves becoming scabrous and uniformly dark on both sides, retaining dense tomentum along veins; base attenuate; margins entire, sinuate or occasionally shallowly 6–8 lobed; apex rounded, mucronate; scarcely armed along midvein beneath; petiole 3–3.5 cm long with few to no prickles. *Inflorescence* a supra-axillary andromonoecious cyme 9–11 cm long, consisting of a basal hermaphrodite flower and a distal group of 3–7 (usually) staminate flowers (the most basal staminate flowers occasionally expressing as hermaphrodite), typically 1–2 staminate flowers open at a time, common peduncle typically 2–5 mm (-20) long, rachis slightly less tomentose than stems. Flowers 5-merous, heterostylous. *Hermaphrodite flower* ca. 2.5 cm below the staminate flowers, opening first; pedicel 2–3 cm long at anthesis, elongating further after fertilization, armed with prickles 2–5 mm long; calyx lobes 5–8 mm long, armed with long, straight prickles and stellate trichomes; corolla 4–5 cm in diameter, dusty purple, rotate; acumens ca. 2 mm; ovary glabrous, ca. 2 mm diameter at anthesis; style 11–12 mm (including capitate stigma), curved; stamens equal; filaments ca. 1 mm; anthers 6 mm long, oblong-lanceolate to somewhat tapered, poricidal at the tips, in a loose anther cone. *Staminate flowers* with pedicels 15–16 mm long, unarmed or with few prickles; calyx lobes 7–8 mm long with a few 3–4 mm weak prickles or prickles absent, the lobes ending with a slender filiform acumen ca. 3 mm long, slightly reflexed; corolla 2.5–4.5 cm in diameter, dusty purple, broadly stellate to rotate; acumens ca. 0.5 mm; stamens of same proportions as in hermaphrodite flower; ovary, style, and stigma vestigial and not exserted beyond the stamens. *Fruit* an ellipsoidal berry 2–2.5 cm long, 1.5–2.2 cm wide, light green with dark green stripes when young, maturing to lemon-yellow with faint brown stripes; flesh firm, the locules 2, not liquid-filled and the internal cavity dry, the fruit wall ca. 0.5 cm thick, the fruits retained on plant after maturation. Fruiting pedicels 3.5–4.5 cm long, deflexed so fruits resting on ground at maturity. Fruiting calyx enclosing and exceeding fruit in early development, eventually covering up to 2/3 of developed fruit, the lobes long-acuminate, blunt-tipped, and weakly reflexing as fruit matures, short stellate-pubescent and armed with sharp spines 2–5 mm long, these single or paired along the calyx sutures, the lobes often fused in a 3+2 or 4+1 arrangement. Mature seeds up to ~100 per fruit, 2.5–3.5 mm, tan to dark brown, finely reticulate.

#### Distribution and ecology.

*Solanum watneyi* is presently known from a small range of localities in the sub-arid, monsoon-influenced, zone of northwestern Northern Territory (Fig. 1) at elevations around 100–150 m. The species is locally abundant along the Bullita Stock Route and nearby areas, but abundance elsewhere is not well known. *Solanum watneyi* appears to be associated, at least in Judbarra, with the “Ridges, Hogbacks, Cuestas, and Structural Plateaux” land unit defined by Brocklehurst et al. (1996), where it occurs in open *Eucalyptus* woodlands. Field collections have been made in well-drained limestone-based soils variously described as being sandy, sandy-loamy, clayey-loamy, and loamy. Collections from 70–80 km east of the Bullita section suggest that the species range extends eastward into the Wanimyn Trust and the landscapes around the Buchanan Highway; and collections from Limbunyah Station hint toward a westward range extending toward the Western Australia border (Fig. 1).

**Figure 2.**
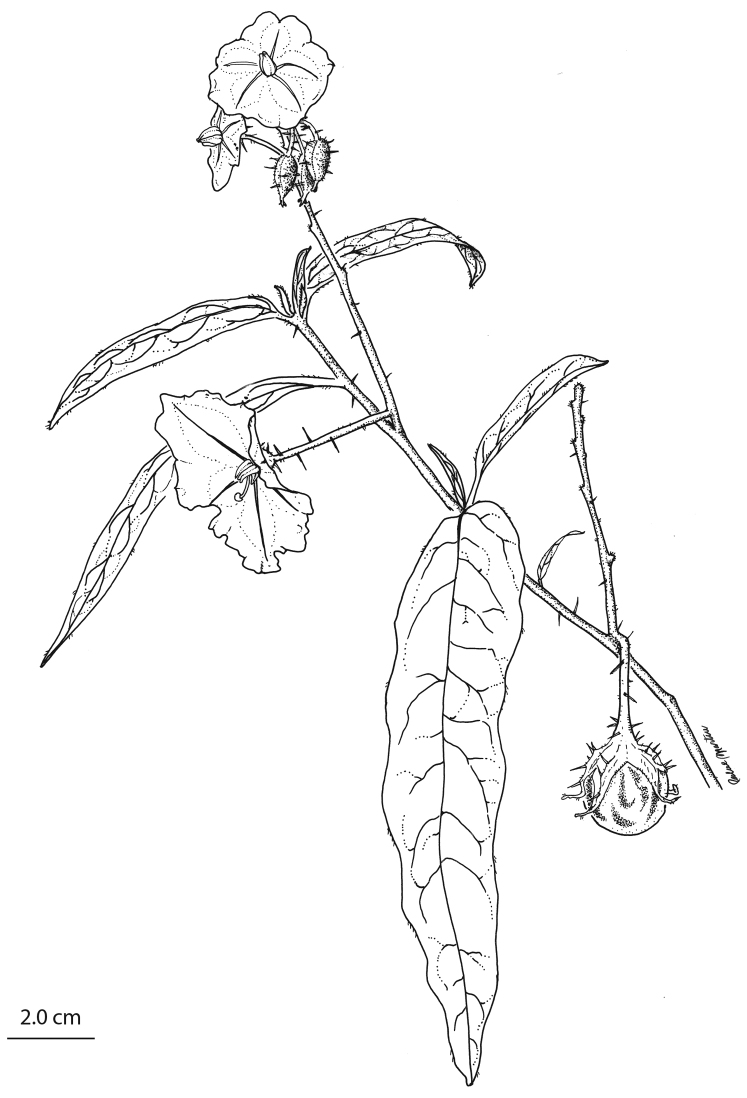
Illustration of *Solanum watneyi*. Mature branch with flowers and a developing fruit. Based on plant grown at Bucknell University from seeds of *Martine and Martine 4065*. Drawing by Rachel F. Martine.

The areas where *Solanum watneyi* has been collected most frequently are along graded roads (Fig. 2D), suggesting that this taxon is disturbance-adapted. Although collections have been made in areas that experience bushfires, specific fire ecology adaptations are unknown. The pollination biology has not been studied, but the flowers are likely buzz pollinated (see Anderson and Symon 1988) – as evidenced by anthers with terminal pores and the need for physical manipulation of anthers during hand pollinations in cultivation. Seed dispersal appears to be biotic given the fleshy nature of the fruits, which ripen to yellow at maturity. Symon (1979) included *Solanum eburneum* in a group of solanums with firm yellow berries likely to be dispersed by mammals or larger birds; *Solanum watneyi* fits this same profile.

**Figure 3.**
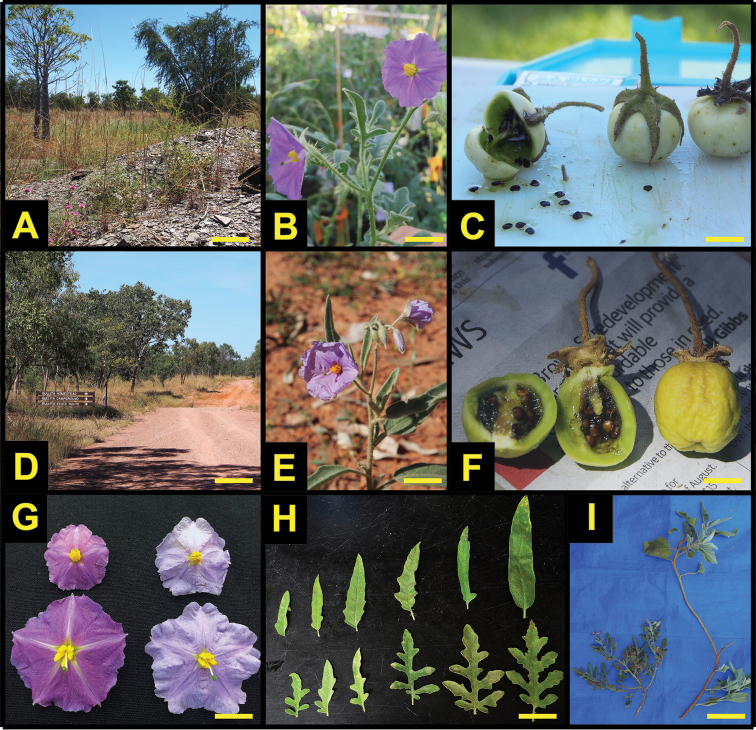
Comparisons of *Solanum eburneum* and *Solanum watneyi*. **A–C** *Solanum eburneum* in habitat (gray cracked clay), in flower, and mature fruits **D–F** same for *Solanum watneyi* (habit showing reddish sandy loam) **G** corolla comparisons of staminate (upper) and hermaphrodite (lower) flowers for *Solanum eburneum* (left) and *Solanum watneyi* (right) **H** leaf shape across varying leaf ages for *Solanum watneyi* (top) and *Solanum eburneum* (bottom) in cultivation; *I*) field growth habit of *Solanum eburneum* (left) and *Solanum watneyi* (right) showing the more sprawling and prostrate nature of *Solanum watneyi*. Photos **A, C, D, E, F, I** by CTM; **B, G** by JC; **H** by EF.

#### Phenology.

Most flowering specimens have been collected from March-May, at the transition from the wet to dry seasons, with fruiting specimens collected in April-June. Earlier flowering collections (e.g. *K. Brennan 9002*, 27 Jan 2000) suggest that blooming may begin during the rainy season and extend into the dry season. Plants in cultivation began flowering about 90 days after seed germination; and fruits took about 60 days to mature following hand pollination.

#### Etymology.

The specific epithet of “watneyi” is inspired by the book and film, *The Martian*, in which the protagonist finds himself stranded on Mars surrounded by the planet’s harsh terrain and reddish soils. In a shelter, he manages to grow a crop of potatoes (*Solanum tuberosum*) before finally being rescued by his astronaut colleagues. We’ve chosen to name *Solanum watneyi* after this character, Mark Watney, in part because of the similarly reddish soils of its habitat and the congeneric nature of the potato – but, most notably, as a way to honor the creation of a sci-fi hero botanist by author Andy Weir (Weir 2013) and to acknowledge perhaps the finest paean to botanical science (and botanical field work) that Hollywood has yet presented (see Martine 2015).

#### Preliminary conservation status.

Based on IUCN Red List Categories (IUCN 2012), *Solanum watneyi* should be considered Data Deficient (DD). While the species appears to be relatively widespread over a range of approximately 6000 km^2^, it has been collected in fewer than 20 localities. The small number of collections, coupled with the fact that populations often consist of multiple individuals, suggests that the species is common in some localities but uncommon on the regional and global scales (apparently of restricted distribution). Further data are required before a certain conservation status can be determined. Key populations are protected in Judbarra/Gregory National Park and appear secure.

#### Specimens examined.

**AUSTRALIA. Northern Territory**: Bullita Homestead (ca. 8 km north), 16°03'--"S, 130°26'--"E, 14 Apr 1996 (fr), *P.K. Latz 14752* (NT); Gregory National Park, Bullock Paddock Creek area, 16°11'21"S, 130°28'59"E, 13 Apr 1996 (fr, fl), *G.J. O’Neill 3* (DNA); Humbert River Station, 16°42'S, 130°40'E, 14 Jun 1974 (fr), P.K. Latz 5325; Bullock Paddock Creek, 16°6'55"S, 130°17'27"E, 13 Apr 1996 (fl), *M. Woodward 94 and R. Booth*; Gregory National Park, N. of Bullita Homestead, 16°06'57"S, 130°25'26"E, 11 Apr 1996 (fl), *N.G. Walsh 4185* (DNA); Gregory National Park, 16.07°S, 130.24°E, 6 Feb 1986 (fl), *B.G. Thomson 1185* (DNA/NT); Jasper Gorge area, 16°1'59"S, 130°42'3"E, 27 Jan 2011(fl), *K. Brennan 9002* (DNA); Judbarra/Gregory National Park, Bullita Stock Route, 32 km south of Victoria Highway, 16°00.491'S, 130°27.952'E, 5 May 2014 (fl, fr), *C.T. Martine and R.F. Martine 4061* (BUPL); Judbarra/Gregory National Park, Bullita campground, 16°06.802'S, 130°25.394'E, 5 May 2014 (juv), *C.T. Martine and R.F. Martine 4063* (BUPL); Judbarra/Gregory National Park, Bullita Stock Route, 6 km north of Bullita Campground turnoff, 16°03.100'S, 130°27.201'E, 6 May 2014 (fl, fr), *C.T. Martine and R.F. Martine* 4065; Judbarra/Gregory National Park, 8 km north of Bullita campground, 16°01.103'S, 130°27.790'E, 5 May 2014 (fl, fr), *C.T. Martine and R.F. Martine 4067* (BUPL).

#### Discussion.

*Solanum watneyi* has been known for some time as a recognized morphotype, having been described by Latz as *Solanum* sp. “Bullita” in the early 1970s. This name has been used as an identifier by other botanists since that time (e.g. Bean 2012), appearing on the labels of herbarium specimens that are similar to *Solanum eburneum* yet conspicuous enough in the characters identified above to merit attention.

Previous studies inferred that *Solanum eburneum* is part of the “Bush tomato clade” (Martine et al. 2006, 2009), a clade of andromonoecious species that appears to have arisen between 4 and 1.5 million years ago (Särkinen et al. 2013). Preliminary results from a large-scale next generation molecular study (Martine et al. in prep) infer that *Solanum watneyi* is most closely related to *Solanum eburneum* and *Solanum chippendalei*. *Solanum watneyi* stands out among this group based on morphological differences that include its conspicuously long fruiting pedicels and internodes, lighter purple corollas, and a sprawling habit that often finds mature fruits resting on ground.

In the western (Bullita) tract of Judbarra/Gregory National Park, *Solanum watneyi* is locally common along the roadsides of the Bullita Stock Route between the Victoria Highway and the old Bullita Homestead, where it has been collected numerous times. Its abundance along this dirt thoroughfare suggests that the species appears, like many solanums, to respond favorably to disturbance. In fact, it appears most vigorously on the edges of graded dirt roads, resprouting from rootstocks after passes from mechanized grading equipment (Martine, pers. obs.)

While our ex situ morphometric analyses and most field observations are consistent with the distinctive morphological gestalt of *Solanum watneyi*, collections from around the Bullita Station can be somewhat confusing – with vegetative characters approaching intermediacy between it and *Solanum eburneum*. A small set of ex situ crosses between them (Martine, unpublished) suggests that interspecific hybridization is possible. Symon (1981) described *Solanum eburneum* as a narrowly-occurring species of “broad, shallow, seasonally dry *Melaleuca* swamps or flats with heavy grey soils” around the East Baines River. This localized endemism aligns with the characteristic physiography described by Brocklehurst et al. (1996) for the East Baines corridor, with *Solanum eburneum* occurring on clays and lithosols found on alluvial plains and rises along the river course as it runs northwest out of the Bullita section of the park (and on Dick Creek to the west and Timber Creek to the east). The presence of *Solanum watneyi* on well-drained sandy-loamy soils on the (largely) southern edge of this range suggest that the two closely related taxa might have diverged on disparate soil types in the last few million years (dating based on Särkinen et al. 2013).

Recent land use history may have brought the species into secondary contact on the north-south edge of their ranges in the central area of the Bullita Section, where an underlying geology including sandstone, siltstone, and dolomite creates an especially complex physiography (Brocklehurst 1996). The remote Bullita cattle station, the homestead of which is now a park attraction and outstation site, operated in this area for several decades beginning in the early 1900s. The Bullita Stock Route, a graded access road running south from the Victoria Highway, closely parallels the East Baines River near the homestead – providing a corridor of frequently disturbed habitat by which *Solanum eburneum* and *Solanum watneyi* might move and come into contact with the aid of road grading equipment or, perhaps historically, livestock. We suggest that the combined effect of ancient-origin physiography and recent disturbance in this area has created a zone of hybridization that deserves further study.

## Supplementary Material

XML Treatment for Solanum watneyi

## Appendix 1

**Specimens examined and mapped (Fig. 1), *Solanum eburneum* Symon. AUSTRALIA. Northern Territory**: Timber Creek, behind caravan park, 15°41'S, 130°31'E, 6 Sep 1993 (fl), *J.R. Lally 114* (DNA); Judbarra/Gregory National Park, Bullita Stn., 15°53'44'S, 130°24'39"E, 13 Feb 1992 (fl), *I. Cowie 2344 and P. Brocklehurst* (DNA); 9 miles west of East Baines River (Dick Creek), Oct 1971 (fl), *D.E. Symon 5232* (DNA); Judbarra/Gregory National Park, Bullita Stock Route, 29 km south of highway, 15°57'57. 4"S, 130°29'06.2"E, 26 May 2004, *C.T. Martine 769 and W.R. Barker* (CONN); Victoria Highway, 22 km west of East Baines crossing, 15°52'10.4"S, 129°52'35.7"E, 26 May 2004 (old fr), *C.T. Martine 770 and W.R. Barker* (CONN); Victoria Highway, East Baines River crossing, 15°45.677'S, 130°01.748'E, 16 April 2014 (fr), *C.T. Martine 4007 and E. Sullivan* (BUPL); Victoria Highway, 5 km east of East Baines River crossing, 15°45.086'S, 130°02.702'E, 16 April 2014 (fr), *C.T. Martine 4007 and E. Sullivan* (BUPL); Bullita Stock Route, 7.5 km south of highway, 15°48.532'S, 130°31.636'E, 5 May 2014 (fl), *C.T. Martine and R.F. Martine 4058* (BUPL); Judbarra/Gregory National Park Bullita Stock Route, 19 km south of highway, 15°54.252'S, 130°30.387'E, 5 May 2014, *C.T. Martine and R.F. Martine 4059* (BUPL); Judbarra/Gregory National Park Bullita Stock Route, 27 km south of highway, 15°58.075'S, 130°29.375'E, 5 May 2014 (fl), *C.T. Martine and R.F. Martine 4060* (BUPL); Judbarra/Gregory National Park Bullita Stock Route, 33 km south of highway, 5 May 2014 (fl), *C.T. Martine 4062 and R.F. Martine 4062* (BUPL).

## References

[B1] Anderson GJ Symon DE (1988) Insect foragers on *Solanum* flowers in Australia. Annals of the Missouri Botanical Garden 75: 842–852. doi: 10.2307/2399372

[B2] Anderson GJ Symon DE (1989) Functional dioecy and andromonoecy in *Solanum*. Evolution 43: 204–219. doi: 10.2307/2409175 10.1111/j.1558-5646.1989.tb04218.x 28568500

[B3] Barrett RL (2013) *Solanum zoeae* (Solanaceae), a new species of bush tomato from the North Kimberley, Western Australia. Nuytsia 23: 5–21. http://florabase.dpaw.wa.gov.au/science/nuytsia/655.pdf

[B4] Bean AR (2012 onwards) *Solanum* species of eastern and northern Australia. Version: 29th June 2013 http://delta-intkey.com

[B5] Bean AR Albrecht DE (2008) *Solanum succosum* A.R.Bean & Albr. (Solanaceae), a new species allied to *S. chippendalei* Symon. Austrobaileya 7: 669–675. http://www.jstor.org/stable/41739087?seq=1#page_scan_tab_contents

[B6] Bean AR (2004) The taxonomy and ecology of Solanum subg. Leptostemonum (Dunal) Bitter (Solanaceae) in Queensland and far north-eastern New South Wales, Australia. Austrobaileya 6: 639–816. http://www.jstor.org/stable/41739063

[B7] Brennan K Martine CT Symon DE (2006) *Solanum sejunctum* (Solanaceae), a new functionally dioecious species from Kakadu National Park, Northern Territory, Australia. The Beagle, Records of the Museums and Art Galleries of the Northern Territory 22: 1–7. http://www.academia.edu/1542271/Solanum_sejunctum_Solanaceae_a_new_functionally_dioecious_species_from_Kakadu_National_Park_Northern_Territory_Australia

[B8] Brocklehurst P Rampant P VanKerckhof D Edmeades B (1996) Vegetation and Land Unit Survey of Gregory National Park, Northern Territory (N.T.). Resource Capability Assessment Branch, Department of Lands, Planning and Environment, Northern Territory Government, Technical Report Number 96/10, Darwin http://www.territorystories.nt.gov.au/bitstream/10070/229353/1/LRD96031.pdf

[B9] IUCN (2012) IUCN Red List Categories and Criteria: Version 3.1. Second edition Gland/Cambridge http://jr.iucnredlist.org/documents/redlist_cats_crit_en.pdf

[B10] Martine CT (2015) Why I’m Naming a New Species after The Martian. Huffington Post 28 September 2015. http://www.huffingtonpost.com/dr-chris-martine/why-im-naming-a-new-plant_b_8190242.html

[B11] Martine CT Vanderpool D Anderson GJ Les DH (2006) Phylogenetic relationship of andromonoecious and dioecious Australian species of Solanum subgenus Leptostemonum section Melongena: Inferences from ITS sequence data. Systematic Botany 31: 410–420. doi: 10.1600/036364406777585801

[B12] Martine CT Anderson GJ Les DH (2009) Gender-bending aubergines: Molecular phylogenetics of cryptically dioecious *Solanum* in Australia. Australian Systematic Botany 22: 107–120. doi: 10.1071/SB07039

[B13] Martine CT Lavoie EM Tippery NP Vogt FD Les DH (2011) DNA analysis identifies *Solanum* from Litchfield National Park as a lineage of *S. dioicum*. Northern Territory Naturalist 23: 29–38. http://www.researchgate.net/publication/235890813_DNA_analysis_identifies_Solanum_from_Litchfield_National_Park_as_a_lineage_of_S._dioicum

[B14] Martine CT Symon DE Capaldi Evans E (2013) A new cryptically dioecious species of bush tomato (*Solanum*) from the Northern Territory, Australia. PhytoKeys 30: 23–31. doi: 10.3897/phytokeys.30.6003 24399898 10.3897/phytokeys.30.6003 PMC3881354

[B15] Särkinen T Bohs L Olmstead RG Knapp S (2013) A phylogenetic framework for evolutionary study of the nightshades (Solanaceae): a dated 1000-tip tree. BMC Evolutionary Biology 13: . doi: 10.1186/1471-2148-13-214 10.1186/1471-2148-13-214 PMC3850475 24283922

[B16] Symon DE (1971) Nine new species of *Solanum*. Transactions and Proceedings of the Royal Society SA 95: 227–239.

[B17] Symon DE (1979) Fruit diversity and dispersal in *Solanum* in Australia. Journal of the Adelaide Botanic Garden 1: 321–331. http://www.jstor.org/stable/23872220

[B18] Symon DE (1981) A revision of genus *Solanum* in Australia. Journal of the Adelaide Botanic Garden 4: 1–367.

[B19] Vorontsova MS Stern S Bohs L Knapp S (2013) African spiny *Solanum* (subgenus *Leptostemonum*, Solanaceae): a thorny phylogenetic tangle. Botanical Journal of the Linnaean Society. doi: 10.1111/boj.12053

[B20] Weir A (2013) The Martian. Published by the author [later published by Crown Publishing Group, New York in 2014]

[B21] Whalen MD (1984) Conspectus of species groups in Solanum subgenus Leptostemonum. Gentes Herbarium 12: 179–282.

